# Amyloid-PET imaging predicts functional decline in clinically normal individuals

**DOI:** 10.1186/s13195-024-01494-9

**Published:** 2024-06-17

**Authors:** Lisa Quenon, Lyduine E. Collij, David Vállez Garcia, Isadora Lopes Alves, Thomas Gérard, Vincent Malotaux, Lara Huyghe, Juan Domingo Gispert, Frank Jessen, Pieter Jelle Visser, Anouk den Braber, Craig W. Ritchie, Mercè Boada, Marta Marquié, Rik Vandenberghe, Emma S. Luckett, Michael Schöll, Giovanni B. Frisoni, Christopher Buckley, Andrew Stephens, Daniele Altomare, Lisa Ford, Cindy Birck, Anja Mett, Rossella Gismondi, Robin Wolz, Sylke Grootoonk, Richard Manber, Mahnaz Shekari, Renaud Lhommel, Laurence Dricot, Adrian Ivanoiu, Gill Farrar, Frederik Barkhof, Bernard J. Hanseeuw, the AMYPAD Consortium

**Affiliations:** 1 Institute of Neuroscience, UCLouvain, Brussels, Belgium; 2 Department of Neurology, Cliniques Universitaires Saint-Luc, Brussels, Belgium; 3 Department of Radiology and Nuclear Medicine, Amsterdam UMC, Vrije Universiteit Amsterdam, Amsterdam, The Netherlands; 4 Amsterdam Neuroscience, Neurodegeneration, Amsterdam, The Netherlands; 5 Clinical Memory Research Unit, Clinical Sciences Malmö, Lund University, Malmö, Sweden; 6 Brain Research Center, Amsterdam, The Netherlands; 7 Department of Nuclear Medicine, Cliniques Universitaires Saint-Luc, Brussels, Belgium; 8 Department of Psychiatry, Massachusetts General Hospital, Harvard Medical School, Charlestown, MA USA; 9 Barcelonaβeta Brain Research Center (BBRC), Pasqual Maragall Foundation, Barcelona, Spain; 10 Centro de Investigación Biomédica en Red de Bioingeniería, Biomateriales y Nanomedicina, Madrid, Spain; 11 Universitat Pompeu Fabra, Barcelona, Spain; 12 Hospital del Mar Medical Research Institute (IMIM), Barcelona, Spain; 13 German Center for Neurodegenerative Diseases (DZNE), Bonn, Germany; 14 Department of Psychiatry, Medical Faculty, University of Cologne, Cologne, Germany; 15 Alzheimer Center Amsterdam, Neurology, Vrije Universiteit Amsterdam, Amsterdam UMC Location VUmc, Amsterdam, The Netherlands; 16 Department of Psychiatry and Neuropsychology, School for Mental Health and Neuroscience, Alzheimer Centrum Limburg, Maastricht University, Maastricht, Netherlands; 17 Biological Psychology, Vrije Universiteit Amsterdam, Amsterdam, The Netherlands; 18 Centre for Clinical Brain Sciences, University of Edinburgh, Edinburgh, UK; 19 Ace Alzheimer Center Barcelona – Universitat Internacional de Catalunya, Barcelona, Spain; 20 Networking Research Center for Neurodegenerative Diseases (CIBERNED), Instituto de Salud Carlos III, Madrid, Spain; 21 Laboratory for Cognitive Neurology, Department of Neurosciences, Leuven Brain Institute, KU Leuven, Louvain, Belgium; 22 Neurology Service, University Hospital Leuven, Louvain, Belgium; 23 Division of Clinical Geriatrics, Center for Alzheimer Research, Department of Neurobiology, Care Sciences and Society, Karolinska Institute, Stockholm, Sweden; 24 Wallenberg Centre for Molecular and Translational Medicine, University of Gothenburg, Göteborg, Sweden; 25 Department of Psychiatry and Neurochemistry, University of Gothenburg, Mölndal, Sweden; 26 Dementia Research Centre, Queen Square Institute of Neurology, University College London, London, UK; 27 Laboratory of Neuroimaging of Aging (LANVIE), University of Geneva, Geneva, Switzerland; 28 Memory Clinic, University Hospital of Geneva, Geneva, Switzerland; 29 GE HealthCare, Pollards Wood, Amersham, UK; 30 Life Molecular Imaging, Berlin, Germany; 31 Neurology Unit, Department of Clinical and Experimental Sciences, University of Brescia, Brescia, Italy; 32 Johnson & Johnson Innovative Medicine, Titusville, NJ USA; 33 Alzheimer Europe, Luxembourg, Luxembourg; 34 GE HealthCare, Glattbrugg, Switzerland; 35 IXICO, London, UK; 36 Queen Square Institute of Neurology and Centre for Medical Image Computing, University College London, London, UK; 37 Gordon Center for Medical Imaging, Department of Radiology, Mass General Brigham, Boston, MA USA; 38 WELBIO Department, WEL Research Institute, Wavre, Belgium

**Keywords:** Amyloid-PET, Centiloid, Preclinical Alzheimer, Functional decline, Instrumental activities of daily living

## Abstract

**Background:**

There is good evidence that elevated amyloid-β (Aβ) positron emission tomography (PET) signal is associated with cognitive decline in clinically normal (CN) individuals. However, it is less well established whether there is an association between the Aβ burden and decline in daily living activities in this population. Moreover, Aβ-PET Centiloids (CL) thresholds that can optimally predict functional decline have not yet been established.

**Methods:**

Cross-sectional and longitudinal analyses over a mean three-year timeframe were performed on the European amyloid-PET imaging AMYPAD-PNHS dataset that phenotypes 1260 individuals, including 1032 CN individuals and 228 participants with questionable functional impairment. Amyloid-PET was assessed continuously on the Centiloid (CL) scale and using Aβ groups (CL < 12 = Aβ-, 12 ≤ CL ≤ 50 = Aβ-intermediate/Aβ± , CL > 50 = Aβ+). Functional abilities were longitudinally assessed using the Clinical Dementia Rating (Global-CDR, CDR-SOB) and the Amsterdam Instrumental Activities of Daily Living Questionnaire (A-IADL-Q). The Global-CDR was available for the 1260 participants at baseline, while baseline CDR-SOB and A-IADL-Q scores and longitudinal functional data were available for different subsamples that had similar characteristics to those of the entire sample.

**Results:**

Participants included 765 Aβ- (61%, *Mdn*_*age*_ = 66.0, *IQR*_*age*_ = 61.0–71.0; 59% women), 301 Aβ± (24%; *Mdn*_*age*_ = 69.0, *IQR*_*age*_ = 64.0–75.0; 53% women) and 194 Aβ+ individuals (15%*, Mdn*_*age*_ = 73.0, *IQR*_*age*_ = 68.0–78.0; 53% women). Cross-sectionally, CL values were associated with CDR outcomes. Longitudinally, baseline CL values predicted prospective changes in the CDR-SOB (*b*_CL*Time_ = 0.001/CL/year, 95% CI [0.0005,0.0024], *p* = .003) and A-IADL-Q (*b*_CL*Time_ = *-*0.010/CL/year, 95% CI [-0.016,-0.004], *p* = .002) scores in initially CN participants. Increased clinical progression (Global-CDR > 0) was mainly observed in Aβ+ CN individuals (*HR*_Aβ+ *vs* Aβ-_ = 2.55, 95% CI [1.16,5.60], *p* = .020). Optimal thresholds for predicting decline were found at 41 CL using the CDR-SOB (*b*_Aβ+ *vs *Aβ-_ = 0.137/year, 95% CI [0.069,0.206], *p* < .001) and 28 CL using the A-IADL-Q (*b*_Aβ+ *vs *Aβ-_ = -0.693/year, 95% CI [-1.179,-0.208], *p* = .005).

**Conclusions:**

Amyloid-PET quantification supports the identification of CN individuals at risk of functional decline.

**Trial registration:**

The AMYPAD PNHS is registered at www.clinicaltrialsregister.eu with the EudraCT Number: 2018-002277-22.

**Supplementary Information:**

The online version contains supplementary material available at 10.1186/s13195-024-01494-9.

## Introduction

Alzheimer’s disease (AD) is assumed to begin with an abnormal accumulation of amyloid-beta (Aβ) proteins in the brain leading to neocortical tau accumulation, cognitive impairment, and functional decline [1]. Functional decline refers to the progressive difficulties that patients experience in performing activities of daily living. Functional impairment is a key defining feature of dementia [2]. However, accumulating evidence has indicated that subtle functional decline may be detectable in the preclinical or asymptomatic stages of AD, when patients perform the instrumental activities of daily living (IADL), namely cognitively complex activities such as cooking, managing medication, or finances, with greater difficulties [3].

To prevent functional decline, disease-modifying therapies are now being tested in asymptomatic individuals with an elevated Aβ load, considered to have preclinical AD [4]. Aβ positron emission tomography (PET) represents the primary method for identifying patients with preclinical AD in many clinical trials [5]. However, the natural history of functional decline in asymptomatic individuals has not yet been comprehensively elucidated. In previous studies, functional outcomes in clinically normal individuals were not consistently associated with the Aβ burden assessed using PET, due in part to methodological differences. The studies that demonstrated an association between Aβ deposition and functional measures generally included very large samples (e.g., > 4000; [6]), participants with subjective cognitive complaints, and/or a follow-up duration of at least 2.4 years [7–10]. In contrast, smaller studies (in either sample or duration) without complainers all failed to provide evidence of an association between Aβ load and functional outcomes [8, 11].

Furthermore, in previous studies, Aβ burden was treated as a binary variable based on specific cutoffs, which may omit critical information at subthreshold values for early detection of at-risk individuals [12], or quantified using the Standard Uptake Value ratio (SUVr), which limits direct result comparisons across studies and translation into clinical practice. The Centiloid (CL) scaling was developed to provide standardized amyloid-PET data on a universal unbounded 0 (mean gray matter signal in healthy young adults) to 100 (mean signal in patients with typical AD) scale, regardless of the radiotracer used [13]. To our knowledge, the CL scale has rarely been used in previous work on the association between Aβ burden and functional decline. By stratifying 534 asymptomatic individuals from the Australian Imaging Biomarkers and Lifestyle study into five groups (< 15 CL = negative, 15–25 CL = uncertain, 26-50 CL = moderate, 51–100 CL = high, > 100 CL = very high), one study showed that progression to Mild Cognitive Impairment (MCI) or dementia at 4.5 years and decline in the Clinical Dementia Rating-Sum of boxes score (CDR-SOB; [14, 15]) were only observable in the CL > 50 groups. While these findings need replication, the authors suggested that the CDR outcome might serve as a relevant endpoint in clinical trials including asymptomatic candidates with a baseline CL > 50 who will be followed for at least 4.5 years, while the therapeutic benefit might be better captured by other metrics in individuals with CL < 50 or a shorter follow-up timeframe [9]. The specific scales that are used for assessing functional abilities properly also represent an important methodological aspect to consider. More nuanced functional scales than the CDR covering a broader range of IADL could help better detect and monitor the subtle functional impairment that may occur early in the Aβ accumulation process.

Therefore, the primary aim of this study was to assess the natural history of functional decline, quantified using both the CDR and a more nuanced IADL scale, and relate it to individuals’ baseline Aβ burden expressed in CL using the Amyloid imaging to prevent Alzheimer’s disease (AMYPAD) Prognostic and Natural History Study (PNHS) database, a large European amyloid-PET dataset phenotyping longitudinally individuals at risk of AD progression [16]. We assumed that the baseline amyloid burden predicts subsequent functional decline in initially clinically normal individuals. More specifically, we expected functional decline in asymptomatic individuals with CL > 50, while we did not exclude the possibility of observing a subtle decline on a nuanced functional scale (i.e., A-IADL-Q) in a group with intermediate baseline CL values (12 ≤ CL ≤ 50). As a secondary objective, we derived CL thresholds optimized to predict functional decline through a data-driven approach.

## Methods

### The Prognostic and Natural History Study (PNHS)

The data used in this article were obtained from the PNHS cohort of the Amyloid imaging to prevent Alzheimer’s disease (AMYPAD) initiative, which aims to evaluate the value of quantitative amyloid-PET measures for predicting progression to AD (for a comprehensive description of the study, see Lopes Alves et al. [17], Bader et al. [18], and the AMYPAD website [19]; dataset version: v202306 [20]). Ten parent cohorts (PCs) contributed to this prospective longitudinal research initiative, across 17 sites spread over seven European countries. All PCs enrolled non-demented older adults at risk of AD-related progression due to their age (i.e., > 50 years). All participants underwent cognitive and functional assessment, an amyloid-PET scan, a 3D T1-weighted magnetic resonance imaging (MRI) and traditional risk factor evaluation (including Apolipoprotein E genotyping, *APOE*). The PNHS recruited 1321 participants between October 2016 and June 2022 [18]. The dataset is accessible upon request on the Alzheimer’s Disease Data Initiative (ADDI) platform [21].

### Standard protocol approvals, registrations, and patient consents

The AMYPAD project was reviewed and approved by the Medical Ethical Committee of the University Medical Center Amsterdam, location VUmc and all local sites. The AMYPAD PNHS is registered on the EU Clinical Trials Register [22] with the EudraCT Number: 2018-002277-22. The study was conducted following the Protocol and the Declaration of Helsinki and Good Clinical Practice. All participants provided written informed consent to participate in this study.

### Participants

Participants were selected from the PNHS cohort if they had the following data available: quantified amyloid-PET at baseline and (a) functional measures within six months from the baseline amyloid-PET for cross-sectional analyses, and (b) longitudinal functional data for prospective analyses.

### Functional measures

#### The Clinical Dementia Rating

The Clinical Dementia Rating (CDR; [14, 15]) assesses six functional domains (i.e., memory, orientation, judgment and problem-solving, community affairs, home and hobbies, and personal care), using five-point scales ranging from 0 to 3 (i.e., 0 = no cognitive impairment, 0.5 = questionable or very mild impairment, 1/2/3 = mild/moderate/severe impairment). The total score, called the CDR-sum of boxes score (CDR-SOB), ranges from 0 to 18, with higher scores indicating greater functional impairment. The global score (Global-CDR) is calculated using an algorithm and is used to characterize clinical progression along the AD spectrum (i.e., 0 = normal, 0.5 = questionable or very mild dementia, 1/2/3 = mild/moderate/severe dementia). Participants with a baseline Global-CDR = 0 were thereafter referred to as being initially clinically normal (CN).

#### The Amsterdam IADL questionnaire

The IADL data that were available in the PHNS database were collected using the Amsterdam Instrumental Activity of Daily Living Questionnaire (A-IADL-Q; [23–25]), an adaptive and informant-based tool covering seven IADL categories (i.e., household activities, household appliances, finances, work, computer use, appliances, leisure activities). Each item is scored on a five-point scale ranging from 'no difficulty in performing the task' to 'no longer able to perform the task'. The total score (T-score) represents the latent trait of ‘daily functioning’ and is normally distributed (*M* = 50, *SD* = 10), with higher scores indicating better IADL functioning.

#### Amyloid-PET imaging

The amyloid-PET acquisition protocols were the same across sites and the scanners were qualified by IXICO before the study started at each site, except for the PCs that entered the PNHS with historical scans. In that case, the historical protocol was maintained to ensure longitudinal consistency. The amyloid-PET imaging procedures used are fully described in Lopes et al. [17] and Collij et al. [16]. Two radiotracers were used in the PNHS, namely [^18^F]florbetaben (NeuraCeq®) and [^18^F]flutemetamol (Vizamyl®), which were supplied by Life Molecular Imaging (LMI) and GE Healthcare (GE), respectively [16].

#### Image acquisition

Amyloid-PET scans were acquired according to the standard protocol for each radiotracer, starting at 90 min post-injection of 300 MBq (± 20%) for [^18^F]florbetaben and 185 MBq (± 10%) for [^18^F]flutemetamol. Images were collected in 4 frames of 5 min each (90 to 110 min post-injection [26, 27]).

#### Image analysis

Amyloid quantification was performed using the fully automated workflow of IXICO, which uses a subject-specific multi-atlas structural MRI segmentation method (i.e., LEAP; [28]) to maximize the accuracy of the Aβ burden quantification at the individual level. PET frames were co-registered, averaged, and aligned to the closest corresponding 3D T1-weighted MRI available from the PCs. The MRI scans were parcellated using the multi-atlas LEAP methodology.

As amyloid-PET data were acquired using different scanners at multiple sites, a standard operational procedure (SOP) was developed in collaboration with EARL (https://earl.eanm.org/), the initiative of the European Association of Nuclear Medicine (EANM) to ensure optimal data harmonization. This SOP was defined based on a preliminary work in the AMYPAD imaging network consisting in the acquisition of Hoffman phantom scans to account for inter-scanner differences [29]. This work identified several indicators of image quality and the necessity of smoothing kernels to achieve an effective resolution of 8 mm.

The global cortical amyloid burden was calculated using the Centiloid (CL) method [30, 31]. Standard Uptake Value ratio images were first created using LEAP parcellation masks with the whole cerebellum as reference region in native space. Subsequently, the global cortical CL values were computed by applying the standard GAAIN target region to pool amyloid-PET data.

These CL values were treated both continuously and categorically in the analyses. For the categorical aspect, we classified participants into three groups according to their baseline CL value: negative group for CL < 12 (Aβ-), “intermediate” group for 12 ≤ CL ≤ 50 (Aβ±), and positive group for CL > 50 (Aβ+). The lower and upper bounds for the intermediate group closely matched the thresholds that were found to exclude the presence of neuritic plaques and best confirmed neuropathological evidence of AD, respectively [31, 32].

### Statistical analyses

All the statistical analyses were conducted using R version 4.2.2.

The closest CDR/A-IADL-Q measurements within six months from the baseline amyloid-PET were considered for baseline functional outcomes.

Group comparisons were computed using χ^2^ or Fisher exact tests for categorical variables, and Mann–Whitney or Kruskall-Wallis tests for continuous variables. Adjustments for multiple comparisons were implemented using the Bonferroni method.

The cross-sectional association between the baseline continuous CL value or CL group and functional outcomes was assessed through generalized linear models (GLMs) including age, sex and *APOE* ε4 carriership as covariates.

Progression on the Global-CDR was analyzed separately for participants with a Global-CDR = 0 (CN) and a Global-CDR = 0.5. Progression from the CN state to the MCI stage was defined by the attainment of a consistent Global-CDR = 0.5 score at the two last visits. Progression to dementia was defined as having a Global-CDR ≥ 1 by the end of the follow-up (FU). Clinically stable participants and converters were then compared using χ^2^ tests and Mann–Whitney tests for demographics and CL measures. Cox proportional hazards analyses were conducted (using the R package “survival”) to evaluate the effect of the baseline CL group and other variables on the clinical progression. Survival corresponded to the time between baseline and progression or the time of the last available visit. The other variables that were introduced in addition to the baseline CL group as predictors corresponded to the demographic and/or global cognitive measures that differed between converters and non-converters.

Moreover, linear mixed-effects (LME) models with random slopes and intercepts were performed (using the “nlme” R package) to assess the longitudinal association between the baseline CL value and subsequent decline in the CDR-SOB and A-IADL-Q scores, including age, sex, education, *APOE* ε4 carriership and follow-up duration in years as covariates.

Finally, inspired by the methodology used in Farrell et al. [5] to derive thresholds for predicting future cognitive decline and Aβ accumulation, we conducted iterative LME models using a range of cut-offs to classify individuals as Aβ+ (i.e., thresholds from 12 to 50 CL by order of 1) to identify the data-driven derived CL value that could optimally detect subsequent decline in the CDR-SOB and A-IADL-Q scores in Aβ+ individuals in comparison to Aβ- participants (CL < 12). The Akaike information criterion (AIC) was used to compare model fits and select the optimal cutoff values. Conditional AICs [33] were also computed but, as they led to the exact same cutoff selection, they are not reported.

## Results

### Participants’ characteristics

In total, 1260 participants, including 1032 CN (baseline Global-CDR = 0) and 228 individuals with a baseline Global-CDR = 0.5, had available quantified amyloid-PET at baseline and functional outcomes (Table 1). Among these participants, 765 were Aβ- (CL < 12; 61%), 301 were Aβ± (12 ≤ CL ≤ 50; 24%) and 194 were Aβ+ (CL > 50; 15%). Aβ+ participants were older (*H*[2] = 113.45, *p-values* < 0.001) and more frequently *APOE* ε4 carriers (*χ*^*2*^[2] = 108.59, *p-values* < 0.001). The Aβ± individuals were also older and more likely *APOE* ε4 carriers than the Aβ- group (*p-values* < 0.001). Aβ+ individuals had lower baseline Mini-Mental State Examination (MMSE) scores (*H*[2] = 51.79, *p-values* < 0.001). The 1260 participants had an available Global-CDR data at baseline while baseline CDR-SOB and A-IADL-Q scores and longitudinal outcomes were available in different subsamples. The characteristics of these subsamples are reported in Supplemental Tables 1 and 2 and are similar to the characteristics of the entire sample.

**Table 1:** Participants’ characteristics depending on the baseline CL group

**Total *N* = 1260**	**Aβ-** (CL < 12) *N* = 765		**Aβ± ** (12 ≤ CL ≤ 50) *N* = 301		**Aβ+ ** (CL > 50) *N* = 194		***p***	***Post-Hoc***
	*Median*	*Q1* – *Q3*	*Median*	*Q1* – *Q3*	*Median*	*Q1* – *Q3*		
Baseline age (years)	66.0	61.0 – 71.0	69.0	64.0 – 75.0	73.0	68.0 – 78.0	< .001	Aβ- < Aβ± < Aβ+
Sex (% females/males)	59/41%		53/47%		53/47%		.084	–
Education (years)	15.0	12.0 – 17.0	15.0	12.0 – 17.0	14.0	12.0 – 17.0	.194	–
*APOE* ε4 carriers (%Yes/No/Missing)	30/70/0%		46/53/1%		68/29/3%		< .001	Aβ- < Aβ± < Aβ+
Baseline MMSE (/30)	29.0	29.0 – 30.0	29.0	28.0 – 30.0	28.0	27.0 – 30.0	< .001	Aβ+ < Aβ± ≈ Aβ-^*^
Baseline Global-CDR (% CDR = 0/CDR = 0.5)	87/13%		82/18%		59/41%		< .001	Aβ+ ≠ Aβ± ≈ Aβ-

*CL* Centiloid

^*^p-value for the difference between Aβ± and Aβ- participants = .097

**Table 2:** Progression to a higher Global-CDR

**Progression in CN participants**					
**Total *N* = 852**	**Stable** *N* = 806		**Converters** *N* = 46		***p***
	*Median*	*Q1*– *Q3*	*Median*	*Q1* – *Q3*	
Baseline age (years)	66.0	61.0 – 71.0	73.0	70.0 – 79.0	< .001
Education (years)	13.0	10.0 – 18.0	15.0	12.0 – 18.0	.110
Sex (% females/males)	57/43%		50/50%		.314
*APOE* ε4 carriers (%Yes/No/Missing)	37.8/61.8/0.4%		39/61/0%		.992
Baseline MMSE (/30)	29.0	29.0 – 30.0	29.0	28.0 – 30.0	.249
FU duration (years)	3.0	2.0 – 4.4	4.9	3.2 – 5.2	< .001
Number of visits	3.0	2.0 – 3.75	5.0	3.0 – 6.0	< .001
** Baseline CL**	**5.6**	**-1.1 – 16.3**	**12.9**	**2.5 – 42.1**	**.007**
** Baseline CL group (Aβ-/Aβ± /Aβ+)**	**68/23/9%**		**50/26/24%**		**.006**
**Progression in Global-CDR = 0.5 participants**					
**Total *N* = 118**	**Non-demented at FU** *N* = 85		**Demented at FU** *N* = 33		***p***
	*Median*	*Q1*– *Q3*	*Median*	*Q1* – *Q3*	
Baseline age (years)	72.0	67.0 – 76.0	76.0	68.0 – 79.0	.044
Education (years)	15.0	13.0 – 17.0	13.0	12.0 – 16.3	.006
Sex (% females/males)	44/56%		58/42%		.244
*APOE* ε4 carriers (%Yes/No/Missing)	43/55/2%		67/30/3%		.026
Baseline MMSE (/30)	29.0	27.0 – 30.0	26.0	25.0 – 27.0	< .001
FU duration (years)	2.4	1.8 – 4.0	3.9	2.1 – 4.4	.042
Number of visits	3.0	2.0 – 4.0	3.0	2.0 – 3.0	.064
** Baseline CL**	**9.80**	**-1.0 – 51.9**	**66.4**	**30.8 – 91.6**	** < .001**
** Baseline CL group (Aβ-/Aβ± /Aβ+)**	**52/22/26%**		**15/21/64%**		** < .001**

Conversion to MCI was defined as having a consistent Global-CDR = 0.5 on the two last visits. Conversion to dementia was defined as having a Global-CDR ≥ 1 by the end of the FU

*CL* Centiloid, *FU* follow-up

### Baseline association between amyloid burden and functional outcomes

#### Global-CDR

The Aβ+ group included more individuals with a Global-CDR = 0.5 at baseline (41%) than the two other groups (*χ*^*2*^[2] = 82.94, *p-values* < 0.001), which included similar proportions of Global-CDR = 0.5 participants (13% in the Aβ- group vs. 18% in the Aβ± group, *p* = 0.117; Table 1, last row). Moreover, the CL values were associated with the Global-CDR in a logistic regression additionally including age, sex and the *APOE* ε4 carriership (*b*_*CL*_ = 0.014, 95% CI [0.009, 0.019], *p* < 0.001). Older age and male sex also contributed to the baseline Global-CDR (*b*_*age*_ = 0.035, 95% CI [0.017, 0.053], *p* < 0.001; *b*_*male sex*_ = 0.489, 95% CI [0.186, 0.793], *p* = 0.002; *b*_*APOE*ε4_ = 0.244, 95% CI [-0.093, 0.579], *p* = 0.154).

##### CDR-SOB

A total of 823 participants had available CDR-SOB and CL data at baseline, including 483 Aβ- (59%), 226 Aβ± (27%), and 114 Aβ+ individuals (14%; Supplemental Table 1).

The Aβ+ group included more individuals with a CDR-SOB > 0 (34%) compared to the Aβ- (14%) and Aβ± (15%) groups (*χ*^*2*^[2] = 26.71, *p-values* < 0.001), which included comparable proportions of participants with a CDR-SOB > 0 (*p* = 1.0).

CL was associated with the CDR-SOB in a GLM additionally including age, sex and *APOE* ε4 status as covariates (*b*_*CL*_ = 0.007, 95% CI [0.0040, 0.010], *p* < 0.001). Age and sex were also associated with the CDR-SOB (*b*_*age*_ = 0.019, 95% CI [0.009, 0.029], *p* < 0.001; *b*_*male sex*_ = 0.179, 95% CI [0.043, 0.316], *p* = 0.010; *b*_*APOE*ε4_ = 0.080, 95% CI [-0.068, 0.227], *p* = 0.290). The same model using the CL group instead of continuous CL indicated that the effect was driven by the Aβ+ group (*b*_Aβ+ *vs* Aβ-_ = 0.547, 95% CI [0.325, 0.769], *p* < 0.001; *b*_Aβ± *vs* Aβ-_ = -0.062, 95% CI [-0.045, 0.248], *p* = 0.449).

##### A-IADL-Q

A-IADL-Q coupled to CL data at baseline were available for 560 individuals: 331 Aβ- (59%), 162 Aβ± (29%), and 67 Aβ+ (12%; Supplemental Table 1).

Aβ+ individuals (*Mdn* = 68.80, *IQR* = 59.30–72.84; *H*[2] = 7.62, *p* = 0.022) had lower A-IADL-Q scores than Aβ- (*Mdn* = 69.69, *IQR* = 68.32–72.94, *p* = 0.016) and Aβ± individuals (*Mdn* = 70.12, *IQR* = 67.95–72.74, *p* = 0.082), while Aβ± and Aβ- groups did not differ (*p* = 1.0).

In a GLM additionally including age, sex and *APOE* ε4 carriership as covariates, the CL value (or CL group) was not associated with the A-IADL-Q score (*b*_*CL*_ = -0.012, 95% CI [-0.029, 0.004], *p* = 0.148). The only predictor of A-IADL-Q was age (*b*_*age*_ = -0.284, 95% CI [-0.334, -0.235], *p* < 0.001).

### Baseline CL predicts functional decline over time

#### Progression to MCI/dementia among the CN participants

Among the 852 CN participants at baseline with longitudinal Global-CDR data (FU duration: 3.4 ± 1.8 years), 37 individuals (4.3%) converted to MCI after a mean of 3.6 ± 1.6 years (FU duration: 4.5 ± 1.5 years), 9 participants (1.1%) converted to dementia after a mean of 3.8 ± 2.3 years (FU duration: 4.0 ± 2.5 years), and 806 (94.2%) remained clinically stable during their FU (3.4 ± 1.7 years). The 46 converters (5.4%) were older at baseline (*p* < 0.001) and had longer FU durations (*p* < 0.001) than stable individuals (Table 2). Progressors had higher baseline CL values (*p* = 0.007) and accordingly included more Aβ+ individuals than stable individuals (*p* = 0.006; Table 2). The conversion rates were 12.8% in Aβ+ , 6.1% in Aβ± and 4.0% in Aβ- individuals. The Cox proportional hazards analysis including age as a covariate showed that Aβ positivity (CL > 50) and older age were associated with increased risk of progression to MCI or dementia (*HR*_Aβ+ *vs* Aβ-_ = 2.55, 95% CI [1.16, 5.60], *p* = 0.020; *HR*_age_ = 1.09, 95% CI [1.06, 1.13], *p* < 0.001). However, having intermediate Aβ burden (12 ≤ CL ≤ 50) was not associated with increased risk of clinical progression over a global mean FU period of 3.4 ± 1.8 years (*HR*_Aβ± *vs* Aβ-_ = 1.69, 95% CI [0.82, 3.47], *p* = 0.153).

#### Progression to dementia among the Global-CDR = 0.5 participants

Among the 118 participants with a Global-CDR = 0.5 at baseline with follow-up data (FU duration: 3.3 ± 2.2 years), 33 individuals (28%) evolved to dementia (Global-CDR ≥ 1) after a mean of 3.9 ± 2.2 years. These participants were older (*p* = 0.044), included more *APOE* ε4 carriers (*p* = 0.026), had lower educational levels (*p* = 0.006), longer FU durations (*p* = 0.042), and lower baseline MMSE score (*p* < 0.001) than non-demented individuals at FU (Table 2).

Participants who progressed to dementia at FU had higher baseline CL values and accordingly included more Aβ+ individuals than non-demented individuals at FU, *p-values* < 0.001. The Cox proportional hazards analysis including age, education, *APOE* ε4 carriership, baseline MMSE score as co-predictors evidenced that Aβ positivity (CL > 50), having intermediate Aβ load (12 ≤ CL ≤ 50), and a lower baseline MMSE score were associated with increased risk of progression to dementia (*HR*_Aβ+ *vs* Aβ-_ = 9.91, 95% CI [2.27, 43.32], *p* = 0.002; *HR*_Aβ± *vs* Aβ-_ = 4.31, 95% CI [1.06, 17.57], *p* = 0.042; *HR*_MMSE_ = 0.45, 95% CI [0.34, 0.61], *p* < 0.001).

##### CDR-SOB

As the number of participants with a Global-CDR = 0.5 at baseline with longitudinal CDR-SOB data was limited (*N* = 42) and the primary goal of this study was to investigate the natural history of functional impairment in CN individuals, we focused analyses on the 531 CN participants (Global-CDR = 0 at baseline) with available longitudinal CDR-SOB (FU duration: 2.7 ± 1.2 years). This group included 330 Aβ- (62%), 149 Aβ± (28%), and 52 Aβ+ individuals (10%). The FU duration was six months shorter in Aβ+ individuals (*Mdn* = 2.1 years in Aβ+ vs. *Mdn* = 2.7 years in Aβ± and Aβ-, *H*[2] = 5.85, *p* = 0.053; Supplemental Table 2).

**Fig. 1:**
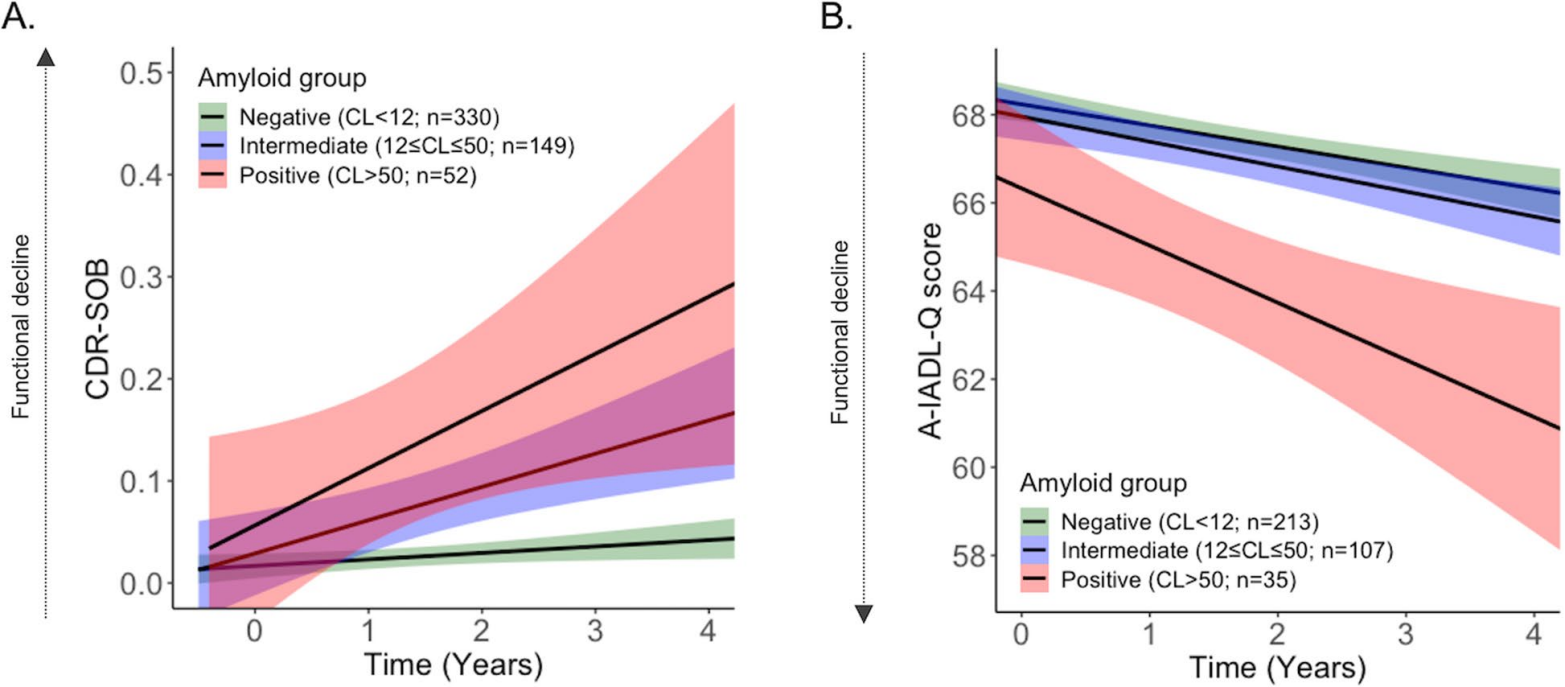
CDR-SOB and A-IADL-Q trajectories over time in CN individuals depending on the baseline CL group. **A** CDR-SOB trajectories in each amyloid group. Higher scores indicate greater functional impairment. **B** A-IADL-Q trajectories in each amyloid group. Lower scores indicate greater functional impairment

The LME predicting the CDR-SOB score over time based on the baseline CL and age highlighted that the baseline CL value predicted prospective changes in the CDR-SOB (*b*_CL*Time_ = 0.001/CL/year, 95% CI [0.0005, 0.0024], *p* = 0.003), while age weakly predicted the CDR-SOB evolution over time (*b*_*age*_ = 0.002/year, 95% CI [-0.0003, 0.0041], *p* = 0.097). Models additionally including sex, education, FU duration, or *APOE* ε4 carriership as covariates did not evidence relevant contributions of these variables to the CDR-SOB changes over time (*p-values* > 0.05). The LME with the CL group, rather than the continuous CL value, showed that the effect was driven by the Aβ+ group (*b*_Aβ+ *vs* Aβ-_ = 0.114/year, 95% CI [0.038, 0.190], *p* = 0.003, *b*_Aβ± *vs* Aβ-_ = 0.034/year, 95% CI [-0.014, 0.082], *p* = 0.160, Fig. 1).

As defined by an increase of 1 point/year [34], the decline on the CDR-SOB score was clinically meaningful in 1.9% (1/52) of Aβ+ individuals, 1.3% (2/149) of Aβ± participants, and 0.3% (1/330) of Aβ- individuals.

##### A-IADL-Q

A total of 355 CN participants had available CL data at baseline and longitudinal A-IADL-Q data (FU duration: 3.0 ± 1.0 years), including 213 Aβ- (60%), 107 Aβ± (30%), and 35 Aβ+ (10%) individuals (no Global-CDR = 0.5 participants at baseline had available longitudinal A-IADL-Q scores). The FU duration differed between groups (*H*[2] = 7.24, *p* = 0.027), being globally five months shorter in Aβ+ individuals (*Mdn* = 2.4 years) than in the two other groups (*Mdn* = 2.9 years; Supplemental Table 2).

The LME predicting A-IADL-Q scores over time based on the baseline CL value and age evidenced that both variables predicted prospective changes on the A-IADL-Q score (*b*_CL*Time_ = *-*0.010/CL/year, 95% CI [-0.016, -0.004], *p* = 0.002; *b*_*age*_ = *-*0.288, 95% CI [-0.329, -0.247], *p* < 0.001). Models additionally including sex, education, FU duration, or *APOE* ε4 status as covariates did not highlight any relevant contributions of these variables to the changes in the A-IADL-Q score over time (*p-values* > 0.05). The LME with CL group, rather than the continuous CL value, as predictor revealed that the effect was driven by the Aβ+ group (*b*_Aβ+ *vs* Aβ-_ = -0.649/year, 95% CI [-1.263, -0.035], *p* = 0.038; *b*_Aβ± *vs* Aβ-_ = -0.225/year, 95% CI [-0.592, 0.141], *p* = 0.227; Fig. 1).

As defined by a loss of 2.2 points on the T-score/year [35], the decline on the A-IADL-Q was clinically meaningful in 11.4% (4/35) of Aβ+ participants, 1.9% (2/107) of Aβ± individuals and 1.9% (4/213) of Aβ- participants.

### Data-driven approach to derive CL thresholds optimally predicting functional decline

**Fig. 2:**
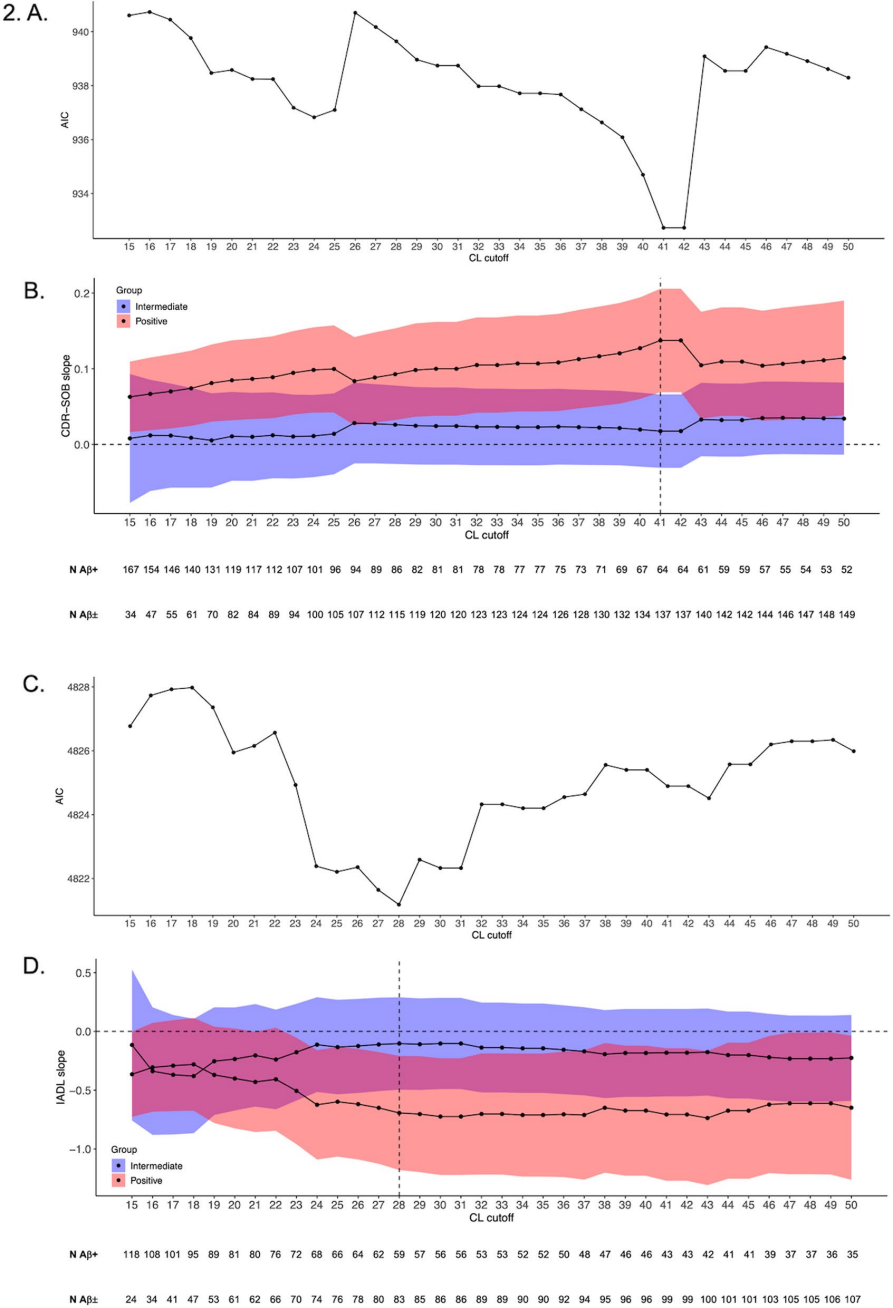
Optimal data-driven thresholds for detecting functional decline on the CDR-SOB and the A-IADL-Q. **A** and **C** The Akaike information criterion (AIC) demonstrating the model fit depending on the tested cutoffs ranging from 15 to 50 CL for the CDR-SOB and the A-IADL-Q, respectively. **B** and **D** CDR-SOB and A-IADL-Q slopes, respectively, vs. Aβ- participants and their 95% CI for the Aβ+ and the Aβ± groups depending on the thresholds used for classifying participants as Aβ+ (participants were classified in the Aβ± group if their baseline CL value was between 12 CL and this cutoff)

The AIC in the iterative LME models using the 15-50 CL range to classify individuals as Aβ+ highlighted that the 41 CL was the lowest value that optimally detected subsequent decline in the CDR-SOB in Aβ+ in comparison to Aβ- participants (Fig. 2A; *b*_Aβ+ *vs* Aβ-_ = 0.137/year, 95% CI [0.069, 0.206], *p* < 0.001). The difference between the slope of the Aβ+ group and the slope of the Aβ- group was maximized at this baseline CL value (Fig. 2B).

The same data-driven approach applied to the A-IADL-Q evidenced that 28 CL optimally detected subsequent decline on this functional outcome in Aβ+ individuals in comparison to the Aβ- group (Fig. 2C, D; *b*_Aβ+ *vs* Aβ-_ = -0.693/year, 95% CI [-1.179, -0.208], *p* = 0.005).

## Discussion

This study aimed to investigate the natural history of functional impairment in a non-demented population using a large European amyloid-PET dataset (AMYPAD; [16]). Cross-sectionally, CL values were associated with CDR outcomes. Longitudinally, baseline CL values predicted prospective changes in the CDR-SOB and A-IADL-Q scores in CN individuals. Over a mean three-year period, increased progression to MCI/dementia and decline in the CDR-SOB and A-IADL-Q scores were only observed in Aβ+ CN individuals (CL > 50). Among participants with a Global-CDR = 0.5 at baseline, progression to dementia over a similar timeframe was increased in both the Aβ+ and Aβ± (12 ≤ CL ≤ 50) groups. Finally, thresholds set at 41 CL and 28 CL optimally predicted a decline in the CDR-SOB score and the A-IADL-Q score, respectively.

While cross-sectional associations between the CL value and CDR-SOB have rarely been described in previous studies, we did not find any association between the IADL measure and the baseline CL value. This finding contradicts the results of previous cross-sectional studies [6, 11, 36]. However, these studies used different IADL scales that, together with other methodological differences, may explain this discrepancy. These methodological differences include the inclusion of more MCI than CN participants in Marshall et al. [11], or the exclusive inclusion of cognitive complainers aged over 70 years old in Lilamand et al. [36]. The only predictor of the A-IADL-Q outcome in our study was age, which is expected with the progressive reduction of functional abilities with aging [37].

Longitudinally, 5.4% of the CN individuals converted to MCI (4.3%) or dementia (1.1%) within 3.4 ± 1.8 years. The converters included more Aβ+ than stable participants. The conversion rate was three times higher in case of amyloid positivity compared to amyloid negativity (12.8% vs. 4.0%). Previous studies reported higher conversion rates in Aβ+ CN participants, ranging from 20-35% (e.g., 32% at 4 years in Donohue et al. [7]; conversion rate of 25% to MCI or dementia over 3.5 ± 1.8 years in Ossenkoppele et al. [38]; 20-32% at 3 years in Papp et al. [39]; 36% at 4.5 years in Sperling et al. [4]). However, these studies used various thresholds to define amyloid positivity and different methods to define progression to MCI (e.g., Petersen’s criteria [40], Global-CDR > 0 without requiring consistent Global-CDR  = 0.5 on several visits for defining reliable progression to MCI). Importantly, in addition to older age and lower education, Aβ positivity (CL > 50) was shown to increase the relative risk of progression from a CN status to MCI or dementia, which is consistent with the previously reported observation that clinical progression in CN after 4.5 years was only observable in the CL > 50 groups [9]. An intermediate amyloid level (12 ≤ CL ≤ 50) was poorly associated with this risk of clinical progression over a global mean FU period of 3.4 ± 1.8 years but longer FU durations may be necessary to significantly increase this risk (e.g., 5.3 ± 1.7 years in 26-50 CL individuals [9]). Moreover, recent studies demonstrated that, in addition to elevated amyloidosis, the regional extent of tau burden appears to accelerate clinical progression in CN [38, 41], suggesting that the coexistence of amyloidosis and regional tau deposition may help in identifying the individuals at higher risk of short-term cognitive decline. Nevertheless, the amyloid levels were binarized in these studies, tagging as Aβ+ the individuals with a baseline CL > 20 [41], which may underestimate the ability of amyloid-PET measurements to efficiently contribute to clinical risk stratification. Head-to-head comparisons of fined-grained PET measurements or staging of both amyloid and tau pathologies need to be conducted to assess their respective value in the clinical risk stratification.

For participants with a Global-CDR = 0.5 at baseline, having an elevated (CL > 50) or intermediate (12 ≤ CL ≤ 50) Aβ burden was associated with an increased risk of progression to dementia after a mean three-year period. The relative risk of progression to dementia was four-fold and nine-fold greater in the Aβ± and Aβ+ groups, respectively, than in the Aβ- group. A lower baseline MMSE score also increased this risk, which is consistent with the findings of previous studies showing that progression to dementia in individuals with MCI is partly conditioned by the extent of initial amyloidosis and cognitive impairment [42]. Moreover, as the risk of clinical progression was increased by an intermediate Aβ burden (12 ≤ CL < 50) in Global-CDR = 0.5 participants but not in CN individuals, it appears that a lower Aβ burden in the former is sufficient to increase the progression risk. This highlights the likely contributions of other factors to functional decline such as other neuropathological changes (e.g., tauopathy), neuroinflammation, cerebrovascular dysfunction, inter-individual differences in terms of cognitive reserve, lifestyle, or genetic risk factors.

Longitudinal analyses of the CDR-SOB and the A-IADL-Q were focused on CN individuals and revealed that the baseline CL value predicted subsequent changes on these metrics, which is consistent with results of previous studies in (partially) CN populations [4, 8–10, 43]. The effect of the CL value was driven by the Aβ+ group for both the CDR-SOB and the A-IADL-Q metrics. No significant decline was detected in the Aβ± group after a mean three-year period. Nevertheless, the iterative LME models using the 15-50 CL range to classify individuals as Aβ+ evidenced that the 41 CL and the 28 CL thresholds optimally detected subsequent decline on the CDR-SOB and the A-IADL-Q, respectively. This suggests that the Aβ± group originally defined as 12 ≤ CL ≤ 50 included some decliners, underscoring the additive value of data-driven methods combined with fine-grained quantification of the Aβ load to fully capture the clinical correlates of amyloidosis [12]. Moreover, while replication and power analyses are needed, these findings also suggest that the CDR-SOB and A-IADL-Q scores may serve as relevant endpoints in 3-year therapeutic trials in asymptomatic individuals with CL values above the abovementioned thresholds.

The fact that the optimal threshold was lower for the A-IADL-Q than for the CDR-SOB may suggest that the former could detect functional decline earlier. This interpretation is also supported by the observation that the decline in the CDR-SOB was subtle and rarely achieved clinically meaningfulness (1.9/1.3/0.3% of Aβ+ /Aβ± /Aβ- individuals). In contrast, a clinically meaningful decline in the A-IADL-Q score was detected in a non-negligible proportion of Aβ+ participants (11.4%) compared to the two other groups (1.9%). However, head-to-head comparisons between these scales should be conducted in larger samples with both outcomes available to address the potential superiority of the A-IADL-Q for monitoring functional decline in a CN population.

The data-driven derived thresholds for the prediction of the subsequent changes on the CDR-SOB and A-IADL-Q scores are higher than the optimal thresholds derived by Farrell et al. [5] for the prediction of cognitive decline in asymptomatic individuals from three different cohorts with comparable FU time-windows (median FU time ranging from two-to-three years), which ranged from 15.0-18.5 CL. This appears consistent with the temporal lag that is assumed between cognitive decline and functional decline [44]. The optimal thresholds derived here also make sense considering the finding that the 26 CL best discriminated participants who would progress to dementia from individuals who would remain clinically stable six years after amyloid-PET in a mixed-population sample of cognitively normal individuals and MCI patients in similar proportions [45]. Moreover, Doré et al. [46] showed that around the 40 CL, there is a steep increase in the prevalence of people with abnormal cortical tau deposition. Clinical progression was found to be faster in individuals with both abnormal amyloid and tau levels (A+ T+) than in individuals with isolated amyloidosis (A+ T-) [38, 41]. It is likely that, in our study, the classification according to the data-driven thresholds grouped individuals who were more likely to have neocortical tau accumulation and therefore a higher risk of functional decline. Functional decline in AD-related diseases is an inherently complex phenomenon that most likely depends on multiple underlying processes. Future studies should test the additive or synergistic contributions of other neuropathological changes such as tauopathy, other proteinopathies, neurodegeneration, neuroinflammation, synaptic dysfunction, cerebrovascular dysfunction, and/or medical comorbidities over larger timescales. This may help better understand the determinants and temporal course of functional decline and improve prognosis at the individual level.

## Limitations

The strengths of this study include the use of a large amyloid-PET dataset with longitudinal clinical follow-up, the inclusion of several functional metrics, the fine-grained quantification of the Aβ load, and the multisite nature of the AMYPAD PNHS project, which, owing to its carefully performed harmonization, ensures the validity and reliability of our findings. However, the participants were predominantly white and highly educated, which limits the generalizability of our findings. Future work should recruit participants with more diverse ethnicities and educational backgrounds. Moreover, this study investigated the natural history of functional impairment depending on the initial amyloid load in the brain. Nevertheless, direct associations between amyloidosis and future functional decline cannot be drawn from the current study. One may assume that the effects of amyloid load on functional decline is most probably indirect, likely involving brain mechanisms that are known to impact cognition and on which amyloidosis has a downstream detrimental effect. Such mechanisms may include tau pathology accumulation, vascular dysfunction, synaptic dysfunction, disrupted functional connectivity, neurotoxicity, and/or inflammatory responses. These mechanisms are interrelated and probably contribute to functional decline sequentially and/or synergistically. Further investigating the contributions of other neuropathological changes (e.g., TDP-43, alpha-synuclein), cognitive reserve, lifestyle, or genetic risk factors is also needed to better understand the complex phenomenon of functional decline.

## Conclusions

While subtle, functional decline over a mean three-year timeframe was observed in CN individuals with elevated amyloid loads as defined by a predefined baseline CL value above 50. However, data-driven approaches suggested that thresholds in the range of CL = 28–41 optimally predicted subsequent functional decline. These findings highlight that the fine-grained quantification of amyloid burden may provide critical information for the prediction of future functional impairment. This may help clinicians to take better decisions for timely preventive interventions (e.g., lifestyle interventions) to postpone functional decline, as long as possible. Moreover, these results support the inclusion of CN individuals with amyloid loads above CL = 28-41 in phase III prevention trials using the A-IADL-Q or CDR-SOB as outcomes as they present an increased risk of short-term functional decline.

### Supplementary Information

Supplementary Material 1.

Supplementary Material 2.

## Data Availability

The dataset is accessible upon request on the Alzheimer’s Disease Data Initiative (ADDI) platform.

## Abbreviations

Aβ: Amyloid-beta
Aβ-: Amyloid-beta negative
Aβ±: Amyloid-beta intermediate
Aβ+: Amyloid-beta positive
AD: Alzheimer’s Disease
ADDI: Alzheimer’s Disease Data Initiative platform
A-IADL-Q: Amsterdam Instrumental Activity of Daily Living Questionnaire
AIC: Akaike Information Criterion
AMYPAD: Amyloid imaging to Prevent Alzheimer’s Disease initiative
APOE: Apolipoprotein E
CDR: Clinical Dementia Rating
CDR-SOB: Clinical Dementia Rating - Sum of boxes
CL: Centiloid
CN: Clinically Normal
EANM: European Association of Nuclear Medicine
FU: Follow-Up
GLM: Generalized Linear Models
Global-CDR: Clinical Dementia Rating - Global score
LME: Linear Mixed Model
MCI: Mild Cognitve Impairment
MMSE: Mini-Mental State Examination
MRI: Magnetic Resonance Imaging
PC: Parent Cohort
PET: Positron Emission Tomography
PNHS: Prognostic and Natural History Study
SOP: Standard Operational Procedure
SUVr: Standard Uptake Value ratio
