# Supplementary material for: Amyloid-PET imaging predicts functional decline in clinically normal individuals
Source: Alzheimers Res Ther. 2024 Jun 17;16:130. doi: 10.1186/s13195-024-01494-9 (PMC11181677; doi:10.1186/s13195-024-01494-9)
Supplement: Supplementary file 1 — Supplementary Material 1. [file 13195_2024_1494_MOESM1_ESM.docx]

**Supplemental Table 1.** Characteristics of participants with available baseline CDR-SOB and A-IADL-Q scores

|  | **Participants with available baseline CDR-SOB (N = 823)** | | | | | | | |
| --- | --- | --- | --- | --- | --- | --- | --- | --- |
|  | **Aβ-**  (CL<12)  N = 483 | | **Aβ±**  (12≤CL≤50)  N = 226 | | **Aβ+**  (CL>50)  N = 114 | | ***p*** | ***Post-Hoc*** |
|  | *Median* | *Q1* – *Q3* | *Median* | *Q1* – *Q3* | *Median* | *Q1* – *Q3* |  |  |
| Baseline age (years) | 65.0 | 60.0 – 70.0 | 68.0 | 63.0 – 73.0 | 73.0 | 67.0 – 76.0 | < .001 | Aβ− < Aβ± < Aβ+ |
| Sex (% women/men) | 58/42% | | 52/48% | | 51/49% | | .165 | – |
| Education (years) | 15.0 | 12.0 – 17.0 | 15.0 | 12.0 – 17.0 | 14.5 | 12.0 – 18.0 | .598 | – |
| APOE ε4 carriers (%Yes/No/Missing) | 33/67/0% | | 48/51/1% | | 69/29/2% | | < .001 | Aβ+ ≠ Aβ± ≠ Aβ− |
| Baseline MMSE (/30) | 30.0 | 29.0 – 30.0 | 29.0 | 28.0 – 30.0 | 29.0 | 27.0 – 30.0 | < .001 | Aβ+ < Aβ± ≈ Aβ− ^*^ |
| Baseline Global-CDR (% CDR=0/CDR=0.5) | 88/12% | | 85/15% | | 67/33% | | < .001 | Aβ+ ≠ Aβ± ≈ Aβ− |
|  | **Participants with available baseline A-IADL-Q score (N = 560)** | | | | | | | |
|  | **Aβ-**  (CL<12)  N = 331 | | **Aβ±**  (12≤CL≤50)  N = 162 | | **Aβ+**  (CL>50)  N = 67 | | ***p*** | ***Post-Hoc*** |
|  | *Median* | *Q1* – *Q3* | *Median* | *Q1* – *Q3* | *Median* | *Q1* – *Q3* |  |  |
| Baseline age (years) | 64.0 | 60.0 – 70.0 | 67.0 | 62.0 – 72.0 | 75.0 | 67.0 – 83.0 | < .001 | Aβ− < Aβ± < Aβ+ |
| Sex (% women/men) | 59/41% | | 56/44% | | 58/42% | | .776 | – |
| Education (years) | 15.0 | 12.0 – 17.0 | 15.0 | 11.0 – 17.0 | 15.0 | 12.0 – 18.0 | .694 | – |
| APOE ε4 carriers (%Yes/No/Missing) | 39/61/0% | | 48/51/1% | | 60/37/3% | | .002 | Aβ+ ≠ Aβ− |
| Baseline MMSE (/30) | 29.0 | 29.0 – 30.0 | 29.0 | 28.0 – 30.0 | 29.0 | 27.0 – 30.0 | < .001 | Aβ+ < Aβ † |
| Baseline Global-CDR (% CDR=0/CDR=0.5) | 94/6% | | 92/8% | | 79/21% | | < .001 | Aβ+ ≠ Aβ± ≈ Aβ− |
| *Note.* CDR-SOB = Clinical Dementia Rating sum of boxes; CL = Centiloid; A-IADL-Q = Amsterdam Instrumental-Activities-of-Daily-Living Questionnaire.  ^*^ *p-value* for the difference between Aβ± and Aβ- participants = .063  † *p-value* for the difference between Aβ± and Aβ- participants = .086; *p-value* for the difference between Aβ± and Aβ+ individuals = .060 | | | | | | | | |
