# Supplementary material for: Amyloid-PET imaging predicts functional decline in clinically normal individuals
Source: Alzheimers Res Ther. 2024 Jun 17;16:130. doi: 10.1186/s13195-024-01494-9 (PMC11181677; doi:10.1186/s13195-024-01494-9)
Supplement: Supplementary file 2 — Supplementary Material 2. [file 13195_2024_1494_MOESM2_ESM.docx]

**Supplemental Table 2.** Characteristics of CN participants at baseline with available longitudinal CDR-SOB and A-IADL-Q scores

|  | **Participants with available FU CDR-SOB (N = 531)** | | | | | | | |
| --- | --- | --- | --- | --- | --- | --- | --- | --- |
|  | **Aβ-**  (CL<12)  N = 330 | | **Aβ±**  (12≤CL≤50)  N = 149 | | **Aβ+**  (CL>50)  N = 52 | | ***p*** | ***Post-Hoc*** |
|  | *Median* | *Q1* – *Q3* | *Median* | *Q1* – *Q3* | *Median* | *Q1* – *Q3* |  |  |
| Baseline age (years) | 64.0 | 60.0 – 69.0 | 67.0 | 62.0 – 72.0 | 71.0 | 65.0 – 77.0 | < .001 | Aβ− < Aβ± < Aβ+ |
| Sex (% females/males) | 57/43% | | 54/46% | | 62/38% | | .649 | – |
| Education (years) | 15.0 | 12.0 – 17.0 | 15.0 | 12.0 – 17.0 | 14.0 | 11.0 – 17.3 | .341 | – |
| APOE ε4 carriers (%Yes/No/Missing) | 37/63/0% | | 48/50/2% | | 65/33/2% | | < .001 | Aβ+ ≈ Aβ± ≠ Aβ− |
| Baseline MMSE (/30) | 29.0 | 29.0 – 30.0 | 29.0 | 28.0 – 30.0 | 29.0 | 28.0 – 30.0 | .018 | Aβ+ ≈ Aβ± ≈ Aβ− ^*^ |
| FU duration (years) | 2.7 | 1.9 – 3.8 | 2.7 | 1.9 – 3.9 | 2.1 | 1.6 – 3.1 | .054 | Aβ+ < Aβ± ≈ Aβ− † |
| Number of visits | 2.0 | 2.0 – 3.0 | 2.0 | 2.0 – 3.0 | 2.0 | 2.0 – 3.0 | .299 | – |
|  | **Participants with available FU A-IADL-Q scores (N = 355)** | | | | | | | |
|  | **Aβ-**  (CL<12)  N = 213 | | **Aβ±**  (12≤CL≤50)  N = 107 | | **Aβ+**  (CL>50)  N = 35 | | ***p*** | ***Post-Hoc*** |
|  | *Median* | *Q1* – *Q3* | *Median* | *Q1* – *Q3* | *Median* | *Q1* – *Q3* |  |  |
| Baseline age (years) | 63.0 | 60.0 – 69.0 | 66.0 | 62.0 – 71.5 | 71.0 | 65.0 – 84.0 | < .001 | Aβ− < Aβ± < Aβ+ |
| Sex (% females/males) | 60/40% | | 53/47% | | 68/32% | | .357 | – |
| Education (years) | 14.0 | 12.0 – 17.0 | 15.0 | 12.0 – 17.0 | 13.0 | 10.0 – 18.0 | .725 | – |
| APOE ε4 carriers (%Yes/No/Missing) | 43/57/0% | | 48/50/2% | | 57/43/0% | | .229 | – |
| Baseline MMSE (/30) | 29.0 | 29.0 – 30.0 | 29.0 | 28.0 – 30.0 | 29.0 | 27.0 – 30.0 | .012 | Aβ+ < Aβ− ‡ |
| FU duration (years) | 2.9 | 2.6 – 3.9 | 2.9 | 2.2 – 4.0 | 2.4 | 1.6 – 3.8 | .027 | Aβ+ < Aβ− § |
| Number of visits | 2.0 | 2.0 – 3.0 | 2.0 | 2.0 – 3.0 | 2.0 | 2.0 – 3.0 | .849 | – |
| *Note.* CDR-SOB = Clinical Dementia Rating sum of boxes; CL = Centiloid; A-IADL-Q = Amsterdam Instrumental-Activities-of-Daily-Living Questionnaire.  ^*^ *p-value* Aβ± vs. Aβ- = .056  † *p-value* Aβ+ vs. Aβ- = .096  ‡ *p-value* Aβ± vs. Aβ- participants = .183; *p-value* Aβ± vs. Aβ+ participants = .384  § *p-value* Aβ± vs. Aβ- participants = .825; *p-value* Aβ± vs. Aβ+ participants = .189 | | | | | | | | |
